# Round about the Asylums.—XII

**Published:** 1888-08-18

**Authors:** 


### Round About the Asylums.—XII.

### WORCESTER COUNTY ASYLUM.

The patients in this asylum increased during the year by 36, and on December 31st there were 877 on the books. Dr. Cooke shows that this increase was due to a lower mortality, and not to an increase of insanity. (There were only 68 deaths during 1887, and during 1886 there were 117.) Indeed, it would seem, from an interesting table printed on page 10 of the report, that pauper insanity has diminished a little in Worcestershire during the last decade relatively to the increase of the population, being 24·6 per 10,000, as against 25·7 for the decade ending 1878, the ratio per 10,000 throughout England and Wales being 25·6 for the last decade. The percentage of recoveries was 34·3 on the admissions, and the deaths were 7·8 on the average number resident. There are 41 private patients, and these pay 15*s.* and 20*s.* a-week, the former being the charge for those belonging to Worcestershire, the latter for outsiders. The weekly rate to the unions is 7*s.* 4*d.* The following remarkable attempt at suicide is recorded: "A young woman, when out walking with a party in the fields, in charge of two nurses, suddenly started off, jumping the gates and fences with such agility that she managed to outstrip the nurse following closely at her heels, entered the garden of a cottage near by, and jumped down a well some thirty feet deep, with five feet of water at the bottom. She was taken out, having fortunately sustained no injury beyond a few scratches. Up to the time of the occurrence she had been in an extremely desponding state; but from that day she began to improve, and is now convalescing." In olden times the plunge bath was a recognised form of treatment in melancholia, and perhaps it may have had its origin in some similar attempt. There were ten escapes, and eight were speedily captured. The Commissioners say: "The means of amusement, in doors and out of doors, continue to be liberally supplied," and they remark on the excellent order of the wards and the thorough knowledge of his patients by the medical superintendents. There are three assistant medical officers. The following appears in the report of the committee of visitors: "Your committee have, through Mr. Hastings, obtained a promise from the Lord Chancellor, that in the Asylum Bill of next session he will insert a clause giving power to committees of visitors to grant to officers and servants, on leaving asylums from confirmed sickness, age, or infirmity, or injury occasioned in the performance of duty, a sum of money down, instead of a retiring pension." We did not notice any such clause in the new Lunacy Bill, nor are pensions of county asylum officers treated in it at all, although the placing of these pensions on a satisfactory basis is a most important matter, involving the whole future welfare of county asylums. Nothing would seem easier than the placing of asylum officers under some modification of the Civil Service Pension Act, but nothing would seem more difficult than getting the framers of the Act to insert the clause.

### WILTS COUNTY ASYLUM, DEVIZES.

This asylum now issues its thirty-seventh annual report. On the first complete year of its existence it contained 219 patients, and it closed the year 1887 with 658, showing an average annual increase of 12; but inasmuch as the population of the county is ever increasing, it is pretty certain that the average increase will in future greatly exceed 12, more especially as it would seem that of late years the increase has not been high. The leeway will have to be made up. The recoveries stand at 29·7 on the admissions, and the deaths at 11 per cent. on the average number resident. The weekly cost per head is as low as 7*s.* 2*d.*—a rate which we think is really too low, and we hope to be able to record a higher one next year. The asylum would seem to be quite full, and it is proposed to build a detached hospital for infectious disease. In such conditions there is always a strong temptation to use these hospitals as ordinary space, and unless vacant beds be kept in the asylum, the hospital is apt to prove a snare and a delusion if required for its legitimate purpose. The Commissioners say that "the condition of the wards deserves praise." The Medical Superintendent's report is extremely clear and well arranged. When speaking of the causes of insanity in those admitted, Dr. Bowes says: "Hereditary influence continues to be the leading cause, and in no less than 49 cases there was a family history of insanity, one female having an epileptic mother and aunt, and an insane grandmother, grandfather, and great-grandfather." Doubtless the disease was hereditary in many more than the 49 cases in which the influence was known to exist, and yet we marry and give in marriage with the most supreme contempt for hereditary disease of all kinds.

### WARNEFORD HOUSE, OXFORD.

This is one of the lunatic hospitals which, to a very large extent indeed, fulfils the object of its creation. It has 77 patients resident, and of these not fewer than 66 have during the last year been kept at various rates below the actual cost per head, which is given at £1 2*s.* 7*d.* per week. On page 18 of the report is a very useful table showing the number of patients treated in the asylum, and the sums paid by them. We learn that five paid as low as 5*s.* a-week; that fourteen were received at 10*s.*; thirteen at 15*s.*, and as already said, there was a total of sixty-six receiving aid. It would seem that two guineas a-week is the highest charge made, an upward limit which it would be well if all hospitals would adopt. We note that thirteen of the patients spent the summer at Eastbourne, and individual cases at other seaside resorts. The Commissioners in Lunacy say that "The hospital is in very good order, and though the weather to-day is severe, we have found the rooms well warmed and comfortable. No seclusion or restraint has been recorded since the last visit." The recoveries stand at the high percentage of 45·5 on the admissions, and the deaths are only 3·8 on the average number resident. The asylum is superintended by Dr. Bywater Ward.

### MIDDLESEX COUNTY ASYLUM AT HANWELL.

One thousand eight hundred and ninety-one patients were resident at the close of the year. Table III. does not give the numbers for each year previous to 1870, but on December 31st of that year there were 1,785 patients in the asylum, so that the average increase for each year was extremely low —a fact doubtless accounted for by the opening of the asylums at Banstead, Leavesden, and Caterham, and also by the fact that there was little vacant space available for increase. The recoveries were 40·60 per cent. on the admissions, and the deaths were 6·46 on the average number resident. The recoveries are a trifle above the average, and the deaths are considerably under the average. Two hundred and ninety-eight patients were admitted, and, as usual, the most potent factors of insanity were hereditary tendency and drink, the former accounting for 87 and the latter for 48. The weekly cost of maintenance was 9*s.* 7½*d.*, a very high rate for so large an asylum. It is well to give prominence to the following remarks from the visitors' report: "It is also becoming a matter of much concern that the provisions of the Criminal Lunatics Act, 1884, are acting unfavourably towards the asylum, in that they tend to largely increase the number of criminal and ex-criminal patients, to the obvious detriment, socially and morally, of the inmates generally." The medical superintendent also comments on this injustice.

